# A Wideband D-Band Frequency Sextupler Chain with High Harmonic Rejection in 100 nm GaAs pHEMT Technology

**DOI:** 10.3390/mi16090984

**Published:** 2025-08-27

**Authors:** Pinqing Wang, Zhe Chen, Yubin Guo, Yue Qi, Peng Yang

**Affiliations:** State Key Laboratory of Millimeter Waves, School of Information Science and Engineering, Southeast University, Nanjing 210096, China; wangpinqing@seu.edu.cn (P.W.); guoyubin@seu.edu.cn (Y.G.); qiyue@seu.edu.cn (Y.Q.); 220220997@seu.edu.cn (P.Y.)

**Keywords:** frequency sextupler, D-band, GaAs, balanced topology

## Abstract

This paper presents a wideband D-band frequency sextupler chain implemented in a 100 nm GaAs pHEMT process. The proposed circuit comprises an input-stage frequency tripler, an inter-stage harmonic-rejection power amplifier, and an output-stage frequency doubler. The tripler adopts a balanced topology, which effectively suppresses the fundamental frequency component. The inter-stage power amplifier not only delivers sufficient drive power to the doubler but also enhances suppression of undesired harmonics. The output doubler employs a single-balanced configuration to suppress odd-order harmonics while extracting the second harmonic. The measured peak output power of the sextupler chain is 2.33 dBm, corresponding to an input power of 2 dBm, resulting in a conversion gain of 0.33 dB. The 3 dB output bandwidth spans from 126.3 to 152.7 GHz, corresponding to a relative bandwidth of 18.9%. Owing to the balanced multiplier topology and harmonic-rejection PA, the 5th and 7th harmonics are suppressed by more than 20 dBc. The combination of high output power, wide operating bandwidth, and excellent harmonic suppression makes the design well suited for wideband D-band signal generation in diverse applications.

## 1. Introduction

With the rapid development of mobile communication technologies, particularly the widespread deployment of 5G and the upcoming 6G systems, spectrum resources are becoming increasingly scarce. Consequently, research into millimeter-wave (mmWave) frequency bands has become increasingly important [1,2]. Within this domain, the D-band has emerged as a key research focus in mmWave communications due to its substantial available bandwidth [3,4].

D-band signal generation is typically realized by cascading a low-frequency source with frequency multipliers [5]. However, undesired harmonic signals generated within the multiplier can degrade the spectral purity of the output. These spurious harmonics are usually suppressed using filters [6]. Nevertheless, in broadband frequency multiplier designs, some undesired harmonics may fall within the target frequency band, rendering conventional filtering less effective.

In this paper, a wideband D-band frequency sextupler chain in a 100 nm GaAs pHEMT process is designed. Both the cascaded frequency tripler and doubler adopt balanced topologies, which inherently suppress unwanted harmonics. A harmonic rejection PA is adopted as well. This configuration eliminates the reliance on additional filters and enhances suitability for broadband applications. The frequency sextupler chain exhibits a peak output power of 2.33 dBm with a 3 dB bandwidth from 126.3 to 152.7 GHz. The circuit topology is given in Section 2 and the measurement results are presented in Section 3.

## 2. Circuit Design

**Figure 1:**
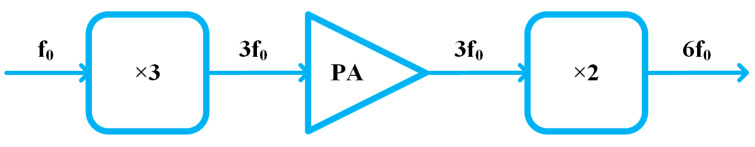
Architecture diagram of the frequency sextupler chain.

As shown in Figure 1, the architecture of the frequency sextupler chain is presented. The tripler is employed as the input stage, while the doubler serves as the output stage. A power amplifier is inserted to provide sufficient drive power for the doubler and to suppress the unwanted harmonics from the tripler.

### 2.1. Design of the Balanced Tripler

**Figure 2:**
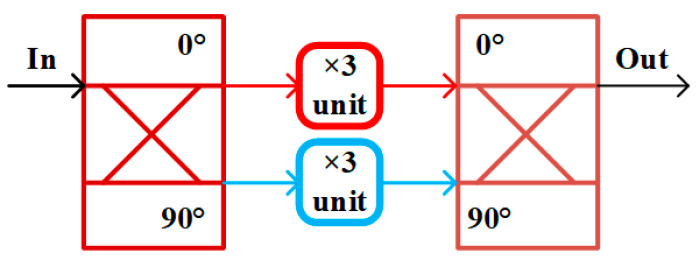
Architecture diagram of the balanced tripler.

The balanced tripler depicted in Figure 2 consists of a fundamental-wave coupler, tripling units, and a third-harmonic coupler [7]. This configuration effectively suppresses the fundamental component and facilitates the extraction of the third harmonic.

In the design, the coupler splits the single-end input signal into two branches with a 90° phase difference. Each branch independently performs the frequency tripling, and their outputs are subsequently combined through the third-harmonic coupler to achieve efficient power summation.

**Figure 3:**
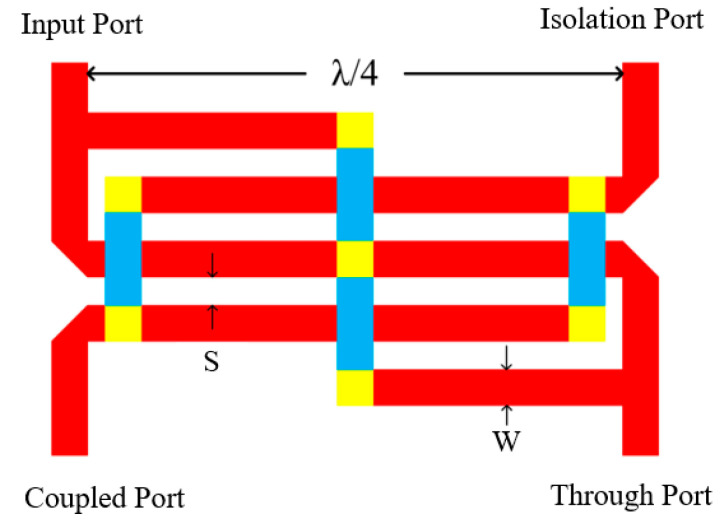
Layout of Lange coupler.

**Figure 4:**
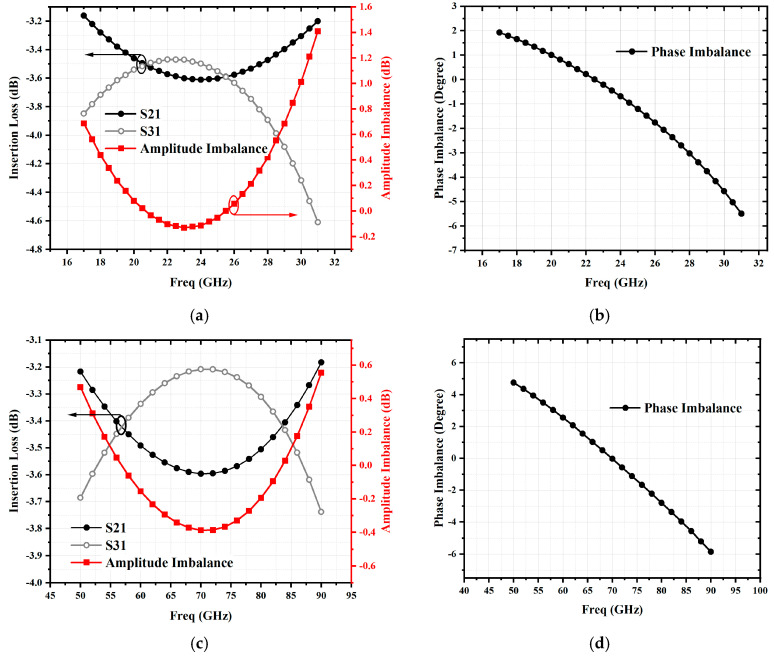
Simulated amplitude and phase imbalance of two Lange couplers: (**a**) amplitude imbalance of fundamental-wave coupler; (**b**) phase imbalance of fundamental-wave coupler; (**c**) amplitude imbalance of third-harmonic coupler; (**d**) phase imbalance of third-harmonic coupler.

As shown in Figure 3, the Lange coupler is composed of several λ/4 transmission line sections. The simulated amplitude and phase imbalances of the two Lange couplers are shown in Figure 4. The amplitude and phase imbalances of the fundamental frequency coupler and the third-harmonic coupler are less than 0.13 dB and 1.5°; 0.4 dB and 4°, respectively.

**Figure 5:**
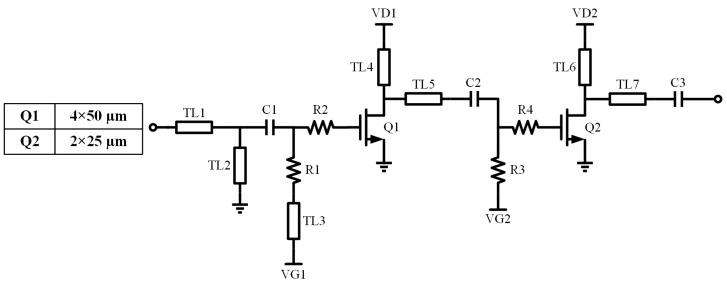
Schematic of tripler unit.

The schematic of the tripler unit is shown in Figure 5, where Q1 serves as a driver amplifier to provide sufficient input power, enabling the tripler transistor Q2 to achieve a high third-harmonic conversion gain. Q2 generates the third harmonic, which is biased in Class A mode to suppress the second harmonics. R1 and R4 are connected in series with the transistor gates to reduce the conversion gain and improve low-frequency stability.

**Figure 6:**
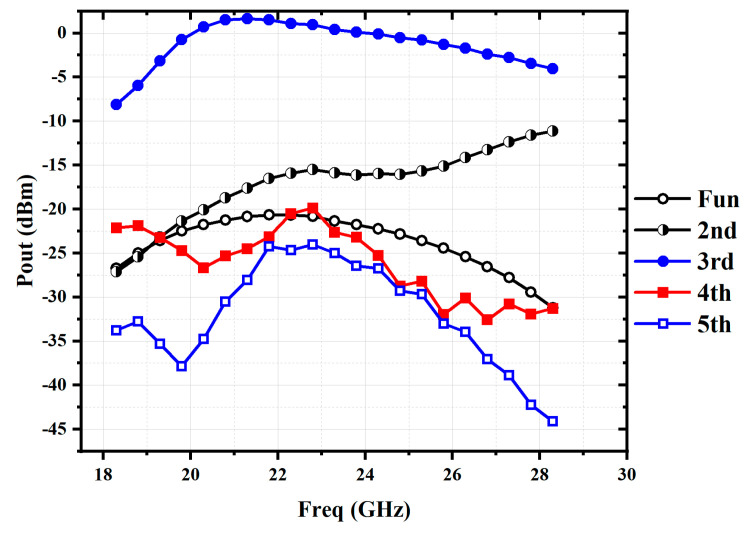
Simulated output power of the balanced tripler with an input level of 0 dBm.

The output power versus the frequency of the proposed balanced tripler is simulated with an input power level of 0 dBm, and the results are shown in Figure 6. The fundamental power is suppressed by more than 20 dBc, and the second harmonic can be further attenuated through post-stage circuit.

### 2.2. Design of the Inter-Stage E-Band PA

**Figure 7:**
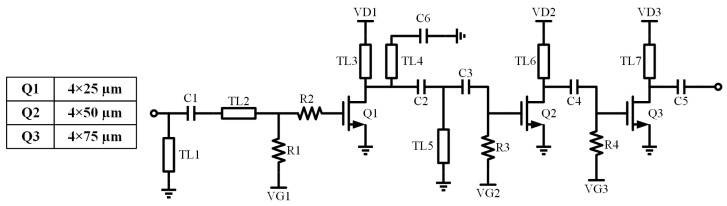
Schematic of PA.

An inter-stage power amplifier is designed to amplify the third-harmonic output of the preceding tripler stage and to provide sufficient drive power for the subsequent passive frequency doubler, as well as adding extra rejection to other unwanted harmonic outputs. This ensures enhanced second-harmonic conversion gain at the output of the doubler. Similar to the tripler, R2 is serially connected to the gate to improve the stability of the PA. The schematic diagram of the designed power amplifier is presented in Figure 7.

**Figure 8:**
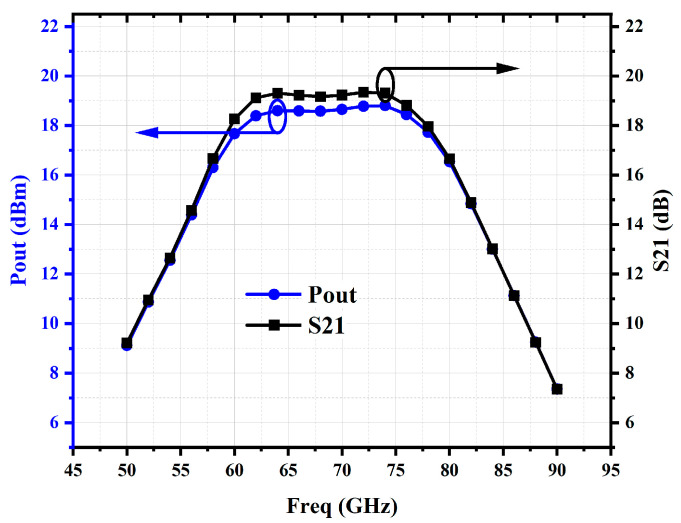
Simulated small-signal gain and large-signal gain of PA.

As shown in Figure 8, the small-signal gain S21 exceeds 19 dB, with a 3 dB bandwidth ranging from 57.7 to 80.3 GHz. Under a 0 dBm input power, the output power exceeds 18 dBm, corresponding to a large-signal gain greater than 18 dB, with a bandwidth extending from 57.2 to 81.1 GHz.

**Figure 9:**
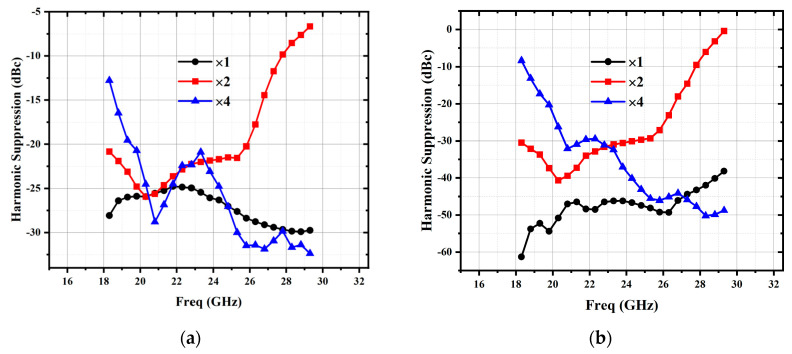
Harmonic suppression of PA: (**a**) harmonic suppression without PA; (**b**) harmonic suppression with PA.

To further suppress the harmonics generated by the preceding tripler stage, the PA matching circuit is designed as a band-pass frequency-selective network. This network is composed of capacitors and microstrip lines, which simultaneously serves the dual purpose of impedance matching and harmonic rejection. As shown in Figure 9, a significant improvement in the suppression of the fundamental, second-order harmonic, and fourth-order harmonic is observed after the PA, from −21.2~−32.5 dBc to −29.6~−50.7 dBc for input frequency of 21.05~25.45 GHz.

### 2.3. Design of the Single-Balanced D-Band Doubler

**Figure 10:**
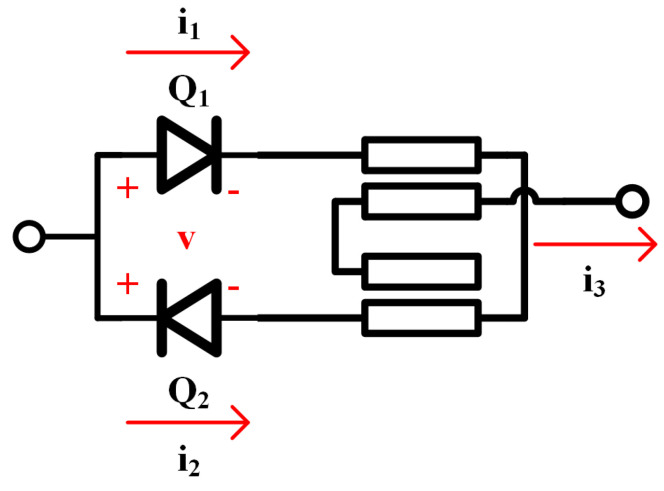
Schematic of single-balanced doubler.

The designed single-balanced doubler consisting of a pair of anti-parallel diodes and a Marchand balun is shown in Figure 10. This architecture effectively utilizes the nonlinear characteristics of diodes to achieve frequency doubling, while suppressing the odd-order harmonics and extracting even-order harmonics [5].

**Figure 11:**
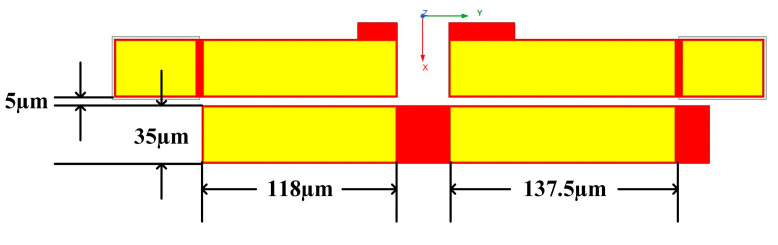
Layout of the asymmetrical Marchand balun.

In the single-balanced doubler, the anti-parallel diodes, which are realized using two discrete diode elements, require input signals of equal amplitude and in-phase relationship to effectively cancel the odd-order harmonics. However, because one diode is driven at the anode and the other at the cathode, this inevitably introduces asymmetry in both the physical structure and the output impedance. The asymmetrical Marchand balun is employed to compensate for this imbalance. The layout is shown in Figure 11; two unequal-length coupled-line sections are utilized for this compensation. All the design parameters are optimized with Advanced Design System.

**Figure 12:**
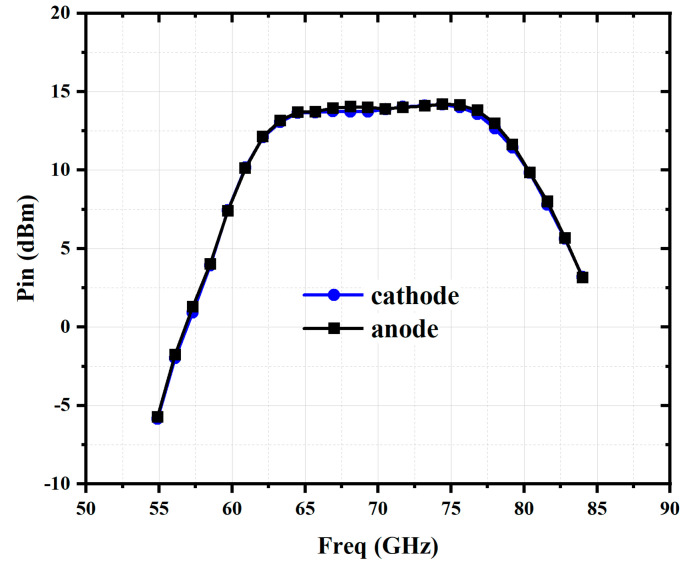
Simulated input power of the single-balanced doubler.

As shown in Figure 12, with this asymmetrical Marchand balun, the two anti-parallel diodes receive equal input power, approximately 14 dBm. In total, the single-balanced doubler obtains an input power of 17 dBm from the PA. All the large-signal simulations were conducted using the harmonic balance with Advanced Design System.

## 3. Measured Results

**Figure 13:**
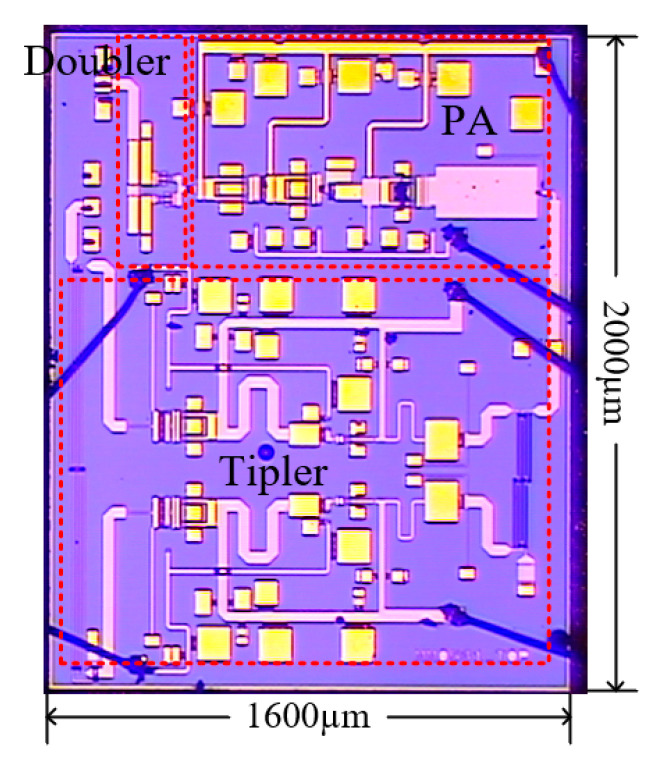
The microphotograph of six-times frequency multiplication chain.

Figure 13 shows a microphotograph of the proposed six-times frequency multiplication chain. The chip occupies a total area of 1600 μm × 2000 μm, including all pads. The circuit is measured using on-wafer probing at room temperature (~26 D.C.). S11 and S22 are measured with GGB probe Model 50A-GSG and Cascade probe I170-T-GSG, using a vector network analyzer (Keysight N5245A) and with VNA extenders (OML V06VNA2-T/R-A_RLA 110-170G), respectively, using SOLT calibration sets. In large-signal measurements, the input signal is provided by a signal generator (R&S SMW200A), while the output power is recorded by an Erickson PM4 power meter and spectrum analyzer (Keysight N9030A) with D-band mixer SFB-06-N1.

**Figure 14:**
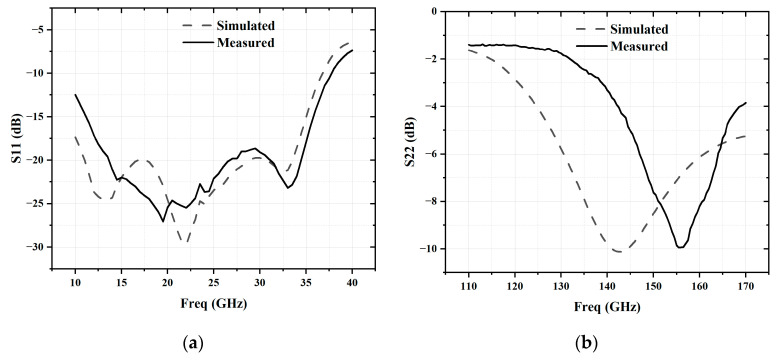
Simulated and measured S-parameters: (**a**) S11; (**b**) S22.

As shown in Figure 14, the measured S11 coincides well with the simulation, remaining below −10 dB across the 10 to 37 GHz frequency range. However, the measured S22 deviates from the simulation, shifting by 13.5 GHz towards higher frequencies. This deviation may be attributed to the fact that the device models in the PDK used in this work are not based on measured data above 90 GHz, but rather derived from curve fitting, which may not be entirely accurate. Meanwhile, the utilized ADS EM simulation tool may also introduce simulation inaccuracies in D-band. And the transistors’ metal figures are added in the EM simulation, which may overestimate the parasitic effects for the passive circuits, leading to a frequency shift in measurement.

**Figure 15:**
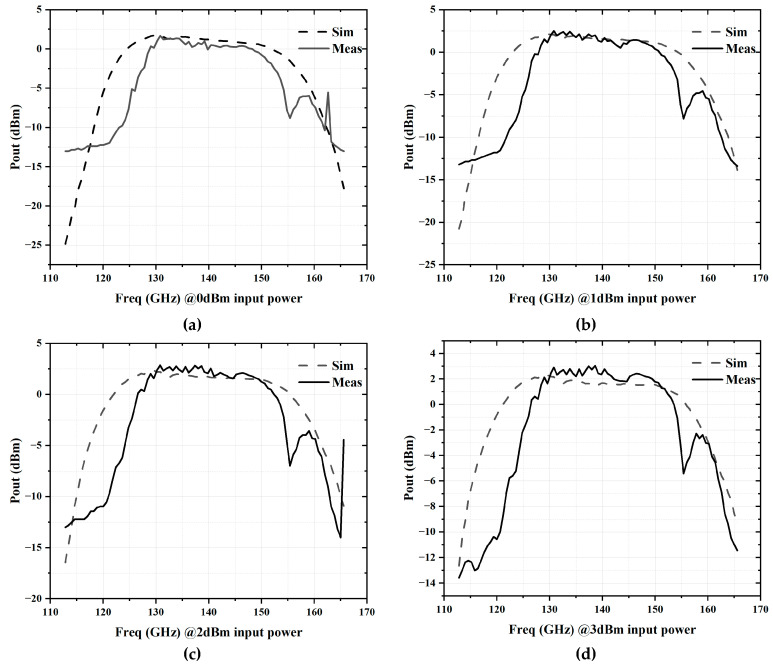
Simulated and measured large-signal versus input power: (**a**) output power @0dBm input power; (**b**) output power @1dBm input power; (**c**) output power @2dBm input power; (**d**) output power @3dBm input power.

Figure 15 shows a comparison between the measured and simulated output power of the frequency sextupler chain under input power levels of 0 dBm, 1 dBm, 2 dBm, and 3 dBm. The measured saturated output power is 3.04 dBm, corresponding to an input power of 3 dBm. At an input power of 1 dBm, the peak output power reaches 2.14 dBm, resulting in a conversion gain of 1.14 dB. The 3 dB output bandwidth spans from 126.9 to 151.8 GHz, giving a relative bandwidth of 17.9%. At an input power of 2 dBm, the peak output power is 2.33 dBm, with a conversion gain of 0.33 dB and an output bandwidth of 3 dB ranging from 126.3 to 152.7 GHz, corresponding to a relative bandwidth of 18.9%.

**Figure 16:**
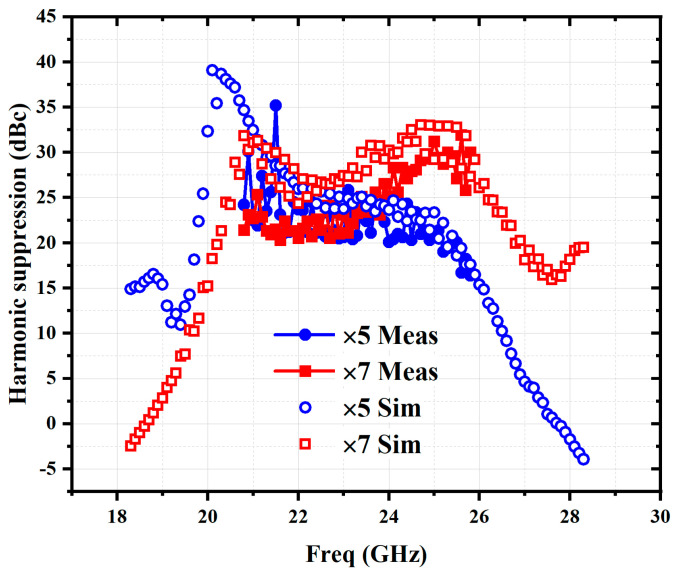
Simulated and measured harmonic suppression.

Figure 16 shows the simulated and measured 5th and 7th harmonic suppression of the frequency sextupler chain at an input power of 2 dBm. The measured results show that within the 3 dB output bandwidth of 126.3–152.7 GHz, both the 5th and 7th harmonics are suppressed by more than 20 dBc. Table 1 compares this work with other reported multiplier designs in GaAs process, where this work shows the widest 3 dB output frequency bandwidth in D-band with high harmonic rejections.

## 4. Conclusions

In this paper, the design of a D-band frequency sextupler chain is presented in a 100 nm GaAs pHEMT process. The presented circuit consists of balanced tripler, harmonic-rejection PA and output doubler. At an input power of 2 dBm, the peak output power of the sextupler reaches 2.33 dBm, resulting in a conversion gain of 0.33 dB. The 3 dB output bandwidth spans from 126.3 to 152.7 GHz, corresponding to a relative bandwidth of 18.9%. Moreover, the 5th and 7th harmonics suppression are better than 20 dBc across the operating frequency range, making it suitable for the wideband signal generation in D-band applications.

## Figures and Tables

**Table 1:** Comparison with other reported GaAs multiplier designs.

Reference	[7]	[8]	[9]	[10]	[11]	[12]	[13]	This Work
Process	100 nm	250 nm	150 nm	100 nm	150 nm	150 nm	100 nm	100 nm
	AlGaAs	GaAs	GaAs	GaAs	GaAs	GaAs	GaAs	GaAs
	mHEMT	pHEMT	pHEMT	pHEMT	pHEMT	pHEMT	pHEMT	pHEMT
Freq (GHz)	79–100	90–99	88–99.5	85–110	34.44–42.56	37–43	71–90	126.3–152.7
Multi Factor	8	3	2	2	2	2	2	6
Gain (dB)	8.9	−3	−4.3	3.2	−4	0.9	−3.3	0.33
Pout (dBm)	6.9	8	7.1	8.2	8	0.9	10.5	2.33
Size (mm^2^)	6	2.21	2	1.35	NA	0.72	1.9	3.2

## Data Availability

The original contributions presented in the study are included in the article, further inquiries can be directed to the corresponding author.
